# Synthesis of [^11^C]BIIB104, an α-Amino-3-hydroxy-5-methyl-4-isoxazolepropionic-Acid-Positive Allosteric Modulator, and Evaluation of the Bio-Distribution in Non-Human Primate Brains Using Positron Emission Tomography

**DOI:** 10.3390/molecules29020427

**Published:** 2024-01-15

**Authors:** Sangram Nag, Kevin Jia, Ryosuke Arakawa, Prodip Datta, Daniel Scott, Christopher Shaffer, Mohammad Mahdi Moein, Matthew Hutchison, Maciej Kaliszczak, Christer Halldin

**Affiliations:** 1 Department of Clinical Neuroscience, Centre for Psychiatry Research, Karolinska Institutet and Stockholm County Council, 171 64 Stockholm, Swedenchrister.halldin@ki.se (C.H.); 2 BIOGEN MA Inc., 225 Binney St., Cambridge, MA 02142, USAchristopher.shaffer@biogen.com (C.S.); matthew.hutchison@biogen.com (M.H.);

**Keywords:** AMPA, radiosynthesis, PET, non-human primate, bio-distribution

## Abstract

The aim of this study was to measure the brain penetrance and kinetics of BIIB104, a first-in-class AMPA receptor potentiator developed for cognitive impairment associated with schizophrenia. It was recently halted in phase 2 clinical development, and there are a lack of tools to directly measure AMPA receptor engagement. To achieve this, the drug candidate was radiolabeled with carbon-11, and its brain penetrance and kinetics were measured in non-human primates via dynamic PET scans. Radiolabeling was achieved through a three-step nucleophilic [^11^C]cyanation reaction in one pot, resulting in the high radioactivity and radiochemical purity (>99%) of [^11^C]BIIB104. The study found that [^11^C]BIIB104 entered the non-human primate brains at 4–5% ID at peak, with a homogeneous distribution. However, a mild regional heterogeneity was observed in the thalamus. The lack of conclusive evidence for a change in regional values after BIIB104 dosing suggests that any specific binding component of BIIB104 is negligible compared to the free and non-specific components in the living brain. Overall, the study demonstrated high brain uptake with minor variability in [^11^C]BIIB104 distribution across various brain regions, its kinetics were consistent with those of passive diffusion, and the dominating components were the free concentration and non-specific binding. This information is valuable for understanding the potential effects and mechanisms of BIIB104 in the brain.

## 1. Introduction

α-Amino-3-hydroxy-5-methyl-4-isoxazolepropionic acid receptors (AMPA receptors) are a subtype of ionotropic glutamate receptors and are primarily activated by the neurotransmitter glutamate, which is released from presynaptic neurons [1]. The structure of AMPA receptors consists of four subunits that form a tetramer. Each subunit consists of an extracellular N-terminal domain, three transmembrane domains (M1, M2, and M3), and an intracellular C-terminal domain [2,3]. The subunits come together to create a transmembrane ion channel, allowing the influx of sodium (Na+) and potassium (K+) ions into the postsynaptic neuron when activated [4,5].

AMPA receptors are widely distributed throughout the brain, with high concentrations found in several key regions. These regions include the hippocampus, the outer cortex, the olfactory area, the lateral septum, and the basal ganglia [6,7,8]. These receptors play a crucial role in mediating fast excitatory synaptic transmissions, which are essential for many neurological processes, including learning, memory formation, and neuronal development [9,10]. The amygdala, another region rich in AMPA receptors, is crucial for processing emotions and fear responses [11]. The discovery and characterization of AMPA receptors have been pivotal in unraveling the mechanisms underlying synaptic plasticity, which forms the basis of learning and memory in the brain. AMPA receptors play a crucial role in facilitating fast excitatory neurotransmission and mediating synaptic signaling. Through the regulation of AMPA receptor function, synaptic strength and efficacy can be modulated, allowing for dynamic changes in synaptic connections and neural circuit activity, ultimately influencing cognitive processes [12,13,14]. This plasticity can be modulated through the regulation of AMPA receptor trafficking and synaptic insertion. Various mechanisms, such as phosphorylation and interactions with auxiliary proteins, can regulate the number and activity of AMPA receptors at a synapse [15,16]. Recent studies have indeed suggested that the dynamic expression of AMPA receptors in the postsynaptic membrane plays a role in various neurological conditions, including schizophrenia [17], epilepsy [18], and Alzheimer’s disease [19]. Understanding the role of AMPA receptor dynamics in these neurological disorders may provide insights into their underlying mechanisms and potentially lead to the development of targeted therapies [20].

PET, which stands for Positron Emission Tomography, is utilized as a molecular imaging method enabling the visualization and measurement of distinct molecular markers in the brain. It serves as a valuable tool in the field of drug discovery for the central nervous system by offering both diagnostic capabilities and the ability to assess the interaction between drugs and targeted molecules [21]. Imaging and quantifying AMPA receptors in the central nervous system (CNS) plays a crucial role in providing valuable insights into the function of these receptors in normal brains and their involvement in different neurological conditions [22]. This process can contribute to a deeper understanding of receptor changes in disease pathology and plays a significant role in drug development and the evaluation of innovative therapies that target AMPA receptors.

Several radiotracers which are designed to bind selectively to AMPA receptors, allowing for the visualization and quantification of receptor distribution and binding affinity, were developed for imaging AMPA receptors using PET [23,24,25,26]. Despite the initial high brain uptake observed in all these tracers during preliminary evaluation, achieving high specific binding in vivo posed a significant challenge [25,27].

BIIB104 (formerly PF-04958242) is a first-in-class AMPA receptor potentiator halted in phase 2 clinical development for cognitive impairment associated with schizophrenia [28,29,30]. However, there have been very limited clinical data confirming AMPA receptor engagement, and tools to directly measure AMPA receptor engagement are lacking. In the absence of a direct method to measure AMPA receptor engagement, a brain bio-distribution study was considered to measure the concentration of BIIB104 in the brain. The aim of this study was (1) to radiolabel BIIB104 with carbon-11, (2) to evaluate the distribution of [^11^C]BIIB104 in non-human primates (NHPs) under baseline conditions and with a pretreatment of a mass dose of BIIB104, and (3) to analyze the radiometabolites in NHP blood plasma.

## 2. Result and Discussion

**Scheme 1:**
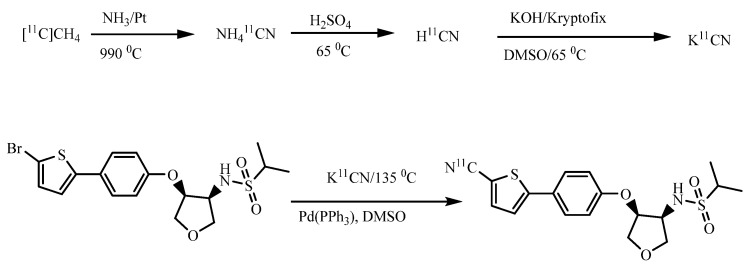
Radiosynthesis of [^11^C]BIIB104.

The entire process of radiosynthesis for [^11^C]BIIB104, which included HPLC purification, SPE isolation, and formulation, took approximately 35 min. The radiosynthesis of [^11^C]BIIB104 was performed according to Scheme 1. The cyanation process using [^11^C]NH_4_CN in three steps was highly reproducible, resulting in over 1500 MBq of the pure final product. This was achieved by irradiating the cyclotron target with a beam current of 35 μA for 15 min. The molar activity (MA) of the produced radioligand ranged from 27 to 49 GBq/µmol at the time of injection into non-human primates (NHPs). The radiochemical purity remained above 99% at the end of the synthesis, and the identity of the radioligand was confirmed by co-injecting it with an authentic reference standard using an HPLC equipped with UV and radio detectors (Figure S1). To ensure stability, the final product, [^11^C]BIIB104, was formulated in a sterile phosphate-buffered solution (PBS) and was found to maintain a radiochemical purity of over 99% for up to 60 min.

[^11^C]Hydrocyanic acid was synthesized using previously published methods [31], with some minor modifications. To summarize the process, [^11^C]CH_4_ was converted in real time within a platinum oven (990 °C) to form [^11^C]ammonium cyanide ([^11^C]NH_4_CN). The resulting [^11^C]NH_4_CN was promptly passed through H_2_SO_4_, leading to the production of [^11^C]HCN. The [^11^C]HCN gas was then guided through a P_2_O_5_ column and introduced into a solution containing Kryptofix_2.2.2_ and KOH dissolved in DMSO, resulting in the formation of [^11^C]KCN. The concentration of NH_3_ gas used plays a crucial role in the formation of [^11^C]HCN, and in our system, we discovered that a concentration of 15% by volume was sufficient. Furthermore, we observed significantly improved conversion rates when the furnace temperature was set to 990 °C.

To enhance the radiosynthesis process, we sought to optimize it by using the corresponding bromo-precursor (BIO-1884126-01). Our optimization efforts involved utilizing two different reaction solvents, namely DMSO and DMF, along with varying amounts (1.0–10.0 mg) of the precursor. We also explored different temperatures to find the ideal conditions for the reaction (Table 1).

After thorough experimentation, we discovered that the best results were obtained when we utilized a precursor quantity of 4.0 mg. Additionally, we found that conducting the reaction in DMSO at a temperature of 135 °C yielded optimal outcomes.

Two female rhesus monkeys, NHP1 and NHP2, were the subjects of a study involving [^11^C]BIIB104, as detailed in Table 2. The injected radioactivity of [^11^C]BIIB104 at the time of injection was measured at 155 ± 6 MBq, with an injected mass of 1.5 ± 0.5 µg.

**Figure 1:**
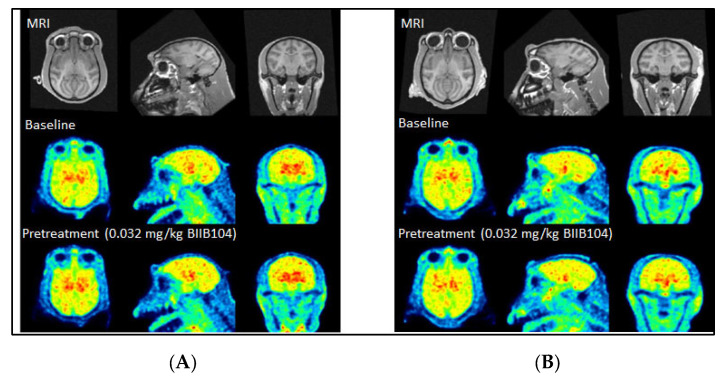
(**A**) PET images in NHP1 after administration of [^11^C]BIIB104 co-registered with MRI in the transaxial (**left**), sagittal (**middle**), and coronal (**right**) projections at baseline (**middle**) and block with BIIB104 (**bottom**). Anatomical reference T1w MRI (**upper**) and (**B**) PET images in NHP2. Images are averages of a total of 93 min just after PET radioligand injection.

**Figure 2:**
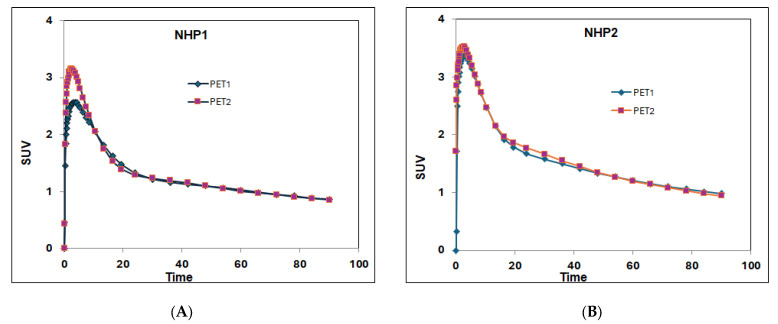
(**A**) Time–activity curves representing the concentration of total radioactivity in NHP 1 brain at baseline (PET 1) and after pretreatment with BIIB (PET 2) and (**B**) that in NHP 2.

**Figure 3:**
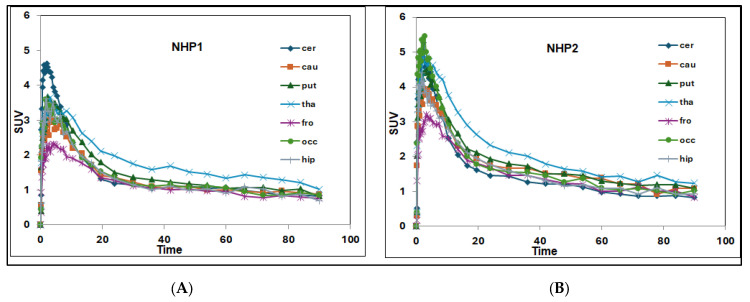
Regional brain uptake of [^11^C]BIIB104 at baseline conditions (**A**) in NHP1 and (**B**) in NHP2.

**Figure 4:**
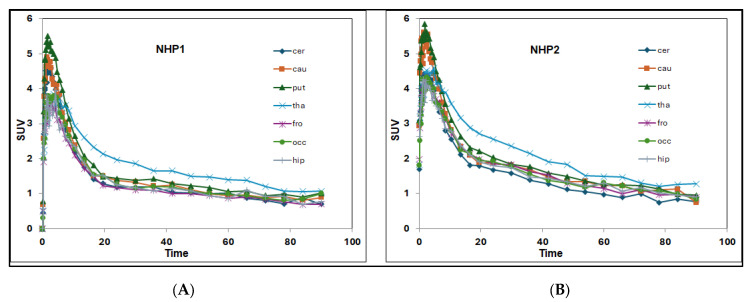
Regional brain uptake of [^11^C]BIIB104 after pretreatment with BIIB104 (**A**) in NHP1 and (**B**) in NHP2.

Summated PET images for the two baseline studies, the two pretreatment studies, and T1w MRI scans for anatomical reference are presented in Figure 1A,B. The whole brain uptake of [^11^C]BIIB104 peaked at 2.6–3.4 SUV (Standardized Uptake Value) under baseline conditions (Figure 2A,B). Initially, a rapid increase in radioligand uptake was observed across the entire brain, with small variations in the SUV among different brain regions. High uptake was observed in the thalamus, followed by the putamen and caudate, and was low in the cortex (Figure 3A,B). The radioligand exhibited swift washout in all brain regions, indicating reversible kinetics for the radioligand. Upon pretreatment with cold BIIB104, there was no significant difference in radioligand uptake, as demonstrated in Figure 4A and 4B, respectively.

For each of the four measurements, regional radioactivity was assessed by analyzing time–activity curves involving the collection of arterial blood samples. These curves were subjected to analysis using both the kinetic 1TC and 2TC models, as well as Logan linear graphical analysis utilizing 60 min data. After evaluating most measurements and brain regions, it was determined that the Logan linear graphical analysis exhibited statistical preference over the 1TC and 2TC models (Table 3A–D). This was because both the 1TC and 2TC models failed to fit accurately in a few regions. After conducting a Logan graphical analysis of VT values obtained at baseline and following the administration of BIIB104, it was determined that there was an 11% decrease in values for NHP1 and a 3% increase in values for NHP2 (Table 4). Generally, brain regionality suggests that the PET radioligand binds to the target specifically, but that of the present study was relatively small. Additionally, VT did not change after pretreatment. These results suggested that [^11^C]BIIB104 may not have sufficient specific binding to AMPA receptors.

**Figure 5:**
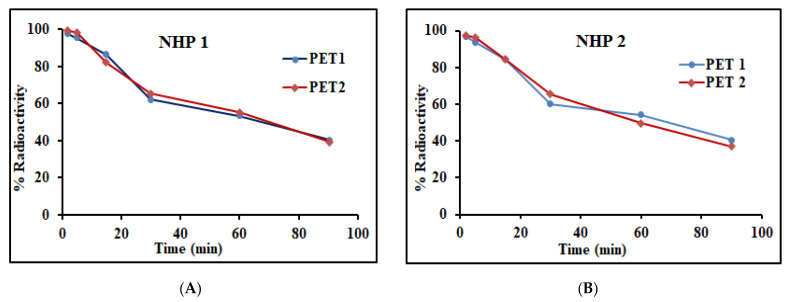
(**A**) The in vivo metabolism of [^11^C]BIIB104 in NHP1 is shown as the relative plasma composition at baseline conditions (PET 1) and after pretreatment with BIIB104 (PET 2). (**B**) The in vivo metabolism of [^11^C]BIIB104 in NHP2 is shown as the relative plasma composition at baseline conditions (PET 1) and after pretreatment with BIIB104 (PET 2).

Following deproteinization, more than 95% of the radioactivity present in plasma was successfully recovered into acetonitrile. HPLC analysis of the plasma after the injection of [^11^C]BIIB104 revealed the elution of the compound from the HPLC column at a retention time of 5.1 min. The parent compound exhibited a higher concentration at 5 min, representing approximately 96% of the eluted compound. Over time, the concentration gradually decreased to about 40% at 90 min for PET under baseline conditions (Figure 5A), and after pretreatment with BIIB104 no significant changes were observed (Figure 5B). Additionally, several peaks of more polar radiometabolites were observed, eluting from the HPLC column before the parent peak. The identity of [^11^C]BIIB104 was confirmed through co-injection with non-radioactive BIIB104.

To evaluate the binding of [^11^C]BIIB104 to monkey plasma proteins, the ultrafiltration method was employed. The results were adjusted based on control samples that accounted for membrane binding. The binding percentages of [^11^C]BIIB104 to monkey plasma proteins were found to be 85% under baseline conditions and 84% after pretreatment with BIIB104. These findings indicate the presence of a measurable free fraction of [^11^C]BIIB104 in plasma, amounting to 15% and 16% under baseline conditions and after pretreatment, respectively.

## 3. Materials and Methods

### 3.1. Radiochemistry

#### General

Liquid chromatographic analysis (LC) was performed with a Merck-Hitachi gradient pump and a Merck-Hitachi L-4000 variable wavelength UV detector. Both the precursor (BIO-1884126-01, *N*-[(3*S*,4*S*)-4-[4-(5-Bromo-2-thienyl)phenoxy]tetrahydrofuran-3-yl]]propane-2-sulfonamide) and the non-radioactive reference standard (BIIB104, *N*-((3*S*,4*S*)-4-[4-(5-Cyano-2-thienyl)phenoxy]tetrahydrofuran-3-yl)propane-2-sulfonamide) were provided by BIOGEN MA Inc, USA. Solid-phase extraction (SPE) cartridges, SepPak C18 Plus, were purchased from Waters (Milford, MA, USA). C-18 Plus cartridge was activated using EtOH (10 mL), followed by using sterile water (10 mL). Radiosynthesis and purification of [^11^C]BIIB104 were performed using a semi-automated custom-made synthesis module. All other chemicals and reagents were bought from commercial sources and used without further purification.

### 3.2. Synthesis of [^11^C]BIIB104

[^11^C]Methane ([^11^C]CH_4_) was produced in-target via the ^14^N(p,α)^11^C reaction on nitrogen mixed with 10% of hydrogen, with 16.4 MeV protons, using a GEMS PET trace cyclotron (GE, Uppsala, Sweden). Typically, the target gas was irradiated for 15–20 min with a beam current of 35 μA. [^11^C]CH_4_ together with NH_3_ (30 mL/min) gas was passed online in a platinum (Pt) oven (carbolite furnace containing platinum wire, at a temperature 990 °C) and was converted to [^11^C]NH_4_CN, which was immediately bubbled through H_2_SO_4_ (50%) at 65 °C to produce [^11^C]HCN. Then, [^11^C]HCN was passed through a P_2_O_5_ column to a reaction vial containing KOH (1 mg) and Kryptofix 2.2.2 (5–6 mg) in DMSO (400 µL) to generate [^11^C]KCN. To the mixture of [^11^C]KCN, bromo-precursor (BIO-1884126-01, 4 mg) and Pd(PPh_3_)_4_ dissolved in dimethyl sulfoxide (DMSO) (300 µL) were added. The reaction mixture was heated at 135 °C for 4 min. The reaction mixture was cooled to room temperature and diluted with sterile water (1 mL) before injecting it into a built-in high performance liquid chromatography (HPLC) system for the purification of the desired radiolabeled product (Figure S2). The HPLC system consisted of a semi-preparative reverse phase (RP) ACE column (C18, 10 × 250 mm, 5 μm particle size) and a Merck Hitachi UV detector (λ = 254 nm) (VWR, International, Stockholm, Sweden) in series with a GM-tube (Carroll-Ramsey, Berkley, CA, USA) used for radioactivity detection. The radioactive fraction corresponding to pure [^11^C]BIIB104 from HPLC was collected and diluted with sterile water (50 mL). The resulting mixture was passed through a pre-conditioned SepPak tC18 plus cartridge. The cartridge was washed with water (10 mL) and the isolated product, [^11^C]BIIB104, was eluted with 1 mL of ethanol. The final purified [^11^C]BIIB104 was formulated in 9 mL phosphate-buffered saline, PBS (pH 7.4), and found to be stable after 1 h. The formulated product was then sterile-filtered through a Millipore Millex^®^ GV filter unit (0.22 μm) for further use in vivo.

### 3.3. Quality Control and Molar Activity (MA) Determination

An analytical HPLC system was employed to determine the radiochemical purity, identity, and stability of the final product, [^11^C]BIIB104. The system consisted of an ACE column (C18, 3.9 Ø × 250 mm, 10 µm particle size), a Merck-Hitatchi L-7100 Pump, an L-7400 UV detector, and a GM-tube for radioactivity detection (VWR International). To elute the product, an HPLC mobile phase of CH_3_CN/TFA (0.1%) in a ratio of 55/45 was used, with a flow rate of 2 mL/min. The effluent was then monitored using a UV absorbance detector (ƛ = 254 nm) coupled with a radioactive detector (b-flow, Beckman, Fullerton, CA). The retention time of [^11^C]BIIB104 was found to be between 3 and 3.5 min. To confirm the identity of [^11^C]BIIB104, it was co-injected with an authentic non-radioactive reference standard.

Furthermore, the final product’s molar activity (MA) was measured using the same analytical HPLC system. The ACE column (C18, 3.9 Ø × 250 mm, 10 µm particle size) was again utilized, but with a mobile phase composition of CH_3_CN/TFA (0.1%) in a ratio of 50/50 and a flow rate of 2 mL/min. The MA was calibrated based on the UV absorbance response per mass of ligand, measured at ƛ = 254 nm. It was calculated by dividing the radioactivity of the radioligand (measured in GBq) by the amount of the associated carrier substance (measured in µmol). 

### 3.4. Study Design and PET Measurements in Non-Human Primates (NHPs)

This study began with the utilization of two female rhesus monkeys (Macaca Mulatta) with respective weights of 6060 g and 6750 g. These non-human primates (NHPs) were housed at the Astrid Fagraeus Laboratory (AFL) of the Swedish Institute for Infectious Disease Control, located in Solna, Sweden. Prior to commencing the study, ethical approval was obtained from the Animal Ethics Committee of the Swedish Animal Welfare Agency. The study followed the guidelines outlined in “Guidelines for planning, conducting and documenting experimental research” (Dnr 4820/06-600) by Karolinska Institutet, as well as the “Guide for the Care and Use of Laboratory Animals” [32].

Four PET measurements were carried out on different experimental days, with each NHP undergoing two PET measurements on the same day. The initial PET measurement involved the administration of [^11^C]BIIB104, while the subsequent measurement took place after pretreatment with cold BIIB104 (0.0032 mg/kg, IV). The pretreatment, BIIB104, was prepared by dissolving it in a mixture of Kolliphor EL (10%) and water (90%). All four PET measurements were conducted using a High-Resolution Research Tomograph (HRRT) scanner (Siemens Molecular Imaging). List-mode data collection lasted for 93 min immediately following the intravenous injection of radioligands. Subsequently, these data were reconstructed using the ordinary Poisson-3D-ordered subset expectation maximization (OP-3D-OSEM) algorithm, which utilized 10 iterations and 16 subsets. Additionally, the reconstruction process included the modeling of the point spread function (PSF). The OP-3D-OSEM PSF achieved an in-plane resolution of 1.5 mm full width at half-maximum (FWHM) at the center of the field of view (FOV) and 2.4 mm at 10 cm off-center directions [33].

The NHPs were induced with anesthesia through intramuscular injection of ketamine hydrochloride (approximately 10 mg/kg) at AFL. The anesthesia was continuously maintained using a mixture of sevoflurane (2.0–8.0%), oxygen, and medical air, with endotracheal intubation taking place in the KI PET center. To ensure immobilization, a fixation device was utilized to secure the NHP heads. Throughout the experiments, body temperature was carefully maintained via a Bair Hugger model 505 (Arizant Healthcare, Eden Prairie, MN, USA) and monitored using an esophageal thermometer. The experiments involved continuous monitoring of vital signs, including ECG, heart rate, blood pressure, respiratory rate, and oxygen saturation. To maintain fluid balance, saline was continuously infused. The imaging process entailed the reconstruction of images through a series of 38 frames, each varying in duration (10 s × 9, 15 s × 2, 20 s × 3, 30 s × 4, 1 min × 4, 3 min × 4, and 6 min × 12). Additionally, a 6 min transmission scan, utilizing a single 137Cs source, was performed prior to each PET acquisition to facilitate attenuation and scatter correction. Brain magnetic resonance imaging was conducted using a 1.5-T GE Signa system (General Electric, Milwaukee, WI, USA).

### 3.5. Arterial Blood Sampling and Radiometabolite Analysis

An Automated Blood-Sampling System (ABSS) was used to measure the continuous radioactivity during the initial 3 min following the injection of the radioligand. However, for the assessment of radioactivity and metabolism at various time points, manual blood sampling was conducted. The analysis of radiometabolites in NHP blood plasma was conducted using a previously published method [34]. Quantitative analysis of [^11^C]BIIB104 and its radioactive metabolites in NHP plasma was performed by employing a simple protein precipitation sample preparation method in combination with reversed-phase radio–HPLC. The chromatographic separation utilized an ACE C18 column with dimensions of 250 mm × 10 mm I.D. A mobile phase consisting of a gradient elution of acetonitrile (A) and 0.1 M ammonium formate (B) was employed at a flow rate of 5.0 mL/min. The gradient conditions were as follows: 0–3.0 min, (A/B) 60:40 → 95:5 *v*/*v*; 3.0–6.0 min, (A/B) 95:5 *v*/*v*. The radio–HPLC system comprised an Agilent binary pump (Agilent 1200 series), a manual injection valve (7725i, Rheodyne) with a 5.0 mL loop, and a radiation detector (Oyokoken, S-2493Z) shielded with 50 mm thick lead for 10 s radio-detector accumulation. Additionally, data collection and control were facilitated using ChemStation Rev. B.04.03 software by Agilent, Stockholm, Sweden.

Following a previously described method [35], after the administration of [^11^C]BIIB104, arterial blood samples were collected from the NHPs at various time points (2, 5, 15, 30, 60, and 90 min). The collected blood was then centrifuged at 4000 rpm for 2 min to obtain plasma. This resulting plasma was diluted with 1.4 times its volume of acetonitrile and subjected to further centrifugation at 6000 rpm for 4 min to separate the extract from the pellet. The supernatant was then diluted with water (3 mL) and injected into a reversed-phase radio–HPLC system.

Within the HPLC system, the eluted radioactive peak corresponding to [^11^C]BIIB104 was integrated, and its area was expressed as a percentage of the total sum of areas of all detected radioactive compounds. To assess the recovery of radioactivity from the system, an aliquot (2 mL) of the eluate from the HPLC column was measured and divided by the total amount of injected radioactivity.

### 3.6. Protein Binding

An ultrafiltration method was employed to measure the free-fraction (fp) of [^11^C]BIIB104 in plasma samples [34]. The procedure began by obtaining a 2 mL blood sample from the NHPs 3–5 min before the radioligand injection. From this, the plasma fraction (400 µL) was separated by centrifugation at 2000 rpm for 2 min. The appropriate amount of formulated radioligand was added to the separated plasma to create a mixture solution (sample A) with a concentration ranging from 1 to 20 MBq/mL. This mixture was then vortexed and left to incubate at room temperature for 10 min. Additionally, a control mixture (sample B) was prepared in a similar manner, substituting the plasma with phosphate-buffered saline (PBS).

Following the incubation, 200 µL portions of samples A and B were pipetted into ultrafiltration tubes (Centrifree YM-30, with a molecular weight cutoff of 30,000 Da; Millipore: Billerica, MA, USA) and subjected to centrifugation at 3800 rpm for 15 min. Equal aliquots (20 µL) of the resulting ultrafiltrate (C_free_) and the original plasma (C_total_) were subjected to radioactivity counting using a NaI well-counter. Each measurement was performed in triplicate. Subsequently, the free fraction of [11C]BIIB104 was calculated as fp = C_free_/C_total_, and the results were adjusted to account for the membrane binding measured using the control samples.

### 3.7. Image and Kinetic Model Analysis

The MRI of each NHP was used to manually delineate the regions of interest (ROIs) for the whole brain, cerebellum, caudate, putamen, thalamus, frontal cortex, occipital cortex, and hippocampus. The summed PET images from the entire scan were then co-registered to the MRI image of each individual NHP. This co-registration allowed for the generation of time–activity curves for brain regions using the dynamic PET data. The main outcome measure involved calculating the total distribution volume (VT), defined as K1/k2 using a one-tissue compartment (1TC) model and (K1/k2) × (k3/k4 + 1) using a two-tissue compartment (2TC) model, as well as using Logan graphical analysis with radiometabolite-corrected plasma radioactivity as the input function. This process provided valuable data for understanding the distribution and kinetics of the drug candidate within specific brain regions.

## 4. Conclusions

The current study successfully demonstrated the efficient labeling of the radioligand [^11^C]BIIB104 with carbon-11 using [^11^C]HCN. PET measurements in rhesus monkeys revealed a substantial uptake of the radioligand in the brain. Interestingly, there were no significant changes observed in brain uptake after pretreatment with cold BIIB104. These results suggest that [^11^C]BIIB104 either may not exhibit strong specific binding to AMPA receptors or we used too low a dose of BIIB104 as pretreatment for a detectable reduction of specific binding.

## Figures and Tables

**Table 1:** Optimization of the radiosynthesis of [^11^C]BIIB104.

Reaction Solvents	Amount of Precursor (mg)	Reaction Temperature (°C)	Incorporation Yield (%)
DMF	0.5	75	0
		135	<1%
		150	<1%
	1.0	100	0
		135	<2%
		150	<2%
	2.0	100	0
		135	<2%
		150	<2%
	5.0	120	3%
		135	7%
		150	5%
	10	120	4%
		135	5%
		150	3%
DMSO	0.5	75	0
		135	5%
		150	4%
	1.0	100	2%
		135	15%
		150	12%
	2.0	120	10%
		135	26%
		150	18%
	4.0	135	57%
	6.0	135	55%
	10.0	135	58%

**Table 2:** Body weight of NHPs, injected radioactivity, mass, and the MA at the time of administration.

NHP	Sex	Body Weight (g)	Injected Radioactivity (MBq)	Molar Radioactivity (GBq/µmol)	Injected Mass (µg)	Condition
NHP1	F	6060	151	49.3	1.20	Baseline
			162	43.7	1.45	BIIB104 (0.032 mg/kg)
NHP2	F	6750	149	49.9	1.17	Baseline
			158	27.0	2.29	BIIB104 (0.032 mg/kg)

**Table 3:** Kinetic parameters and calculation of V_T_ using three different models, 1-TC, 2-TC, and Logan. A. NHP1 at baseline condition, B. NHP1 after pretreatment with BIIB104, C. NHP2 at baseline condition, and D. NHP2 after pretreatment with BIIB104.

A (NHP1 at Baseline)									
	1TC			2TC					Logan
	K1	k2	VT	K1	k2	k3	k4	VT	VT
cer	0.48	0.17	2.89	0.61	0.51	0.36	0.20	3.33	4.01
cau	0.25	0.07	3.36	0.26	0.09	0.01	0.01	4.89	3.90
put	0.30	0.08	3.85	0.31	0.09	0.01	0.01	4.86	4.23
tha	0.29	0.06	4.84	0.31	0.09	0.03	0.04	5.45	5.23
fro	0.18	0.06	3.17	0.19	0.07	0.01	0.02	3.89	3.41
occ	0.31	0.10	3.22	0.33	0.13	0.02	0.03	4.04	3.86
hip	0.30	0.09	3.20	0.31	0.12	0.01	0.02	4.07	3.73
B (NHP1 after pretreatment with BIIB104)									
	1TC			2TC					Logan
	K1	k2	VT	K1	k2	k3	k4	VT	VT
cer	0.51	0.18	2.75	0.54	0.23	0.02	0.14	2.64	3.32
cau	0.53	0.18	2.87	0.68	0.64	0.52	0.25	3.26	3.58
put	0.60	0.19	3.25	0.65	0.24	0.02	0.05	3.69	3.95
tha	0.33	0.07	4.53	0.36	0.11	0.04	0.08	4.80	4.59
fro	0.35	0.13	2.76	0.36	0.15	0.01	0.03	3.26	3.12
occ	0.37	0.12	3.06	0.38	0.14	0.01	0.01	4.16	3.41
hip	0.35	0.12	2.95	0.39	0.20	0.07	0.09	3.37	3.32
C (NHP2 at baseline)									
	1TC			2TC					Logan
	K1	k2	VT	K1	k2	k3	k4	VT	VT
cer	0.48	0.19	2.56	0.73	1.16	1.06	0.32	2.68	2.96
cau	0.33	0.11	3.05	0.36	0.17	0.04	0.06	3.62	3.50
put	0.38	0.12	3.24	0.41	0.17	0.03	0.05	3.83	3.71
tha	0.40	0.10	3.97	0.53	0.49	0.60	0.21	4.22	4.22
fro	0.24	0.08	2.87	0.26	0.11	0.02	0.05	3.11	3.05
occ	0.51	0.18	2.78	0.68	0.67	0.53	0.29	2.92	3.37
hip	0.37	0.14	2.67	0.59	0.86	0.66	0.19	3.01	3.16
D (NHP2 after pretreatment with BIIB104)									
	1TC			2TC					Logan
	K1	k2	VT	K1	k2	k3	k4	VT	VT
cer	0.37	0.15	2.51	0.53	0.95	1.20	0.33	2.59	2.95
cau	0.47	0.17	2.73	0.67	0.78	0.61	0.25	3.00	3.55
put	0.49	0.16	3.00	0.67	0.63	0.48	0.23	3.25	3.77
tha	0.33	0.08	4.02	0.45	0.59	0.87	0.19	4.30	4.28
fro	0.33	0.12	2.87	0.37	0.19	0.06	0.08	3.38	3.34
occ	0.32	0.11	2.93	0.34	0.15	0.02	0.04	3.56	3.39
hip	0.33	0.12	2.76	0.39	0.20	0.07	0.09	3.37	3.32

**Table 4:** Determination of VT using Logan linear graphical analysis in NHP1 and NHP2.

NHP1	Logan	NHP2	Logan
cer	−17%	cer	0%
cau	−8%	cau	+2%
put	−6%	put	+2%
tha	−12%	tha	+1%
fro	−8%	fro	+10%
occ	−12%	occ	+1%
hip	−11%	hip	+6%
Average	−11%		+3%

## Data Availability

All the supporting data are stored at Karolinska Institutet’s archive.

## Supplementary Materials

The following supporting information can be downloaded at: https://www.mdpi.com/article/10.3390/molecules29020427/s1, Figure S1: Analytical HPLC chromatogram for the QC of [11C]BIIB104 BIIB104. The upper represents the UV and the lower represents the radio chromatogram. Figure S2: Semi-preparative HPLC chromatogram for the purification of [11C]BIIB104. Upper represents the UV, the middle represents the radio chromatogram and the bottom represents the fraction corresponds to [^11^C]BIIB104 collected from HPLC.
