# Supplementary material for: Synthesis of [11C]BIIB104, an α-Amino-3-hydroxy-5-methyl-4-isoxazolepropionic-Acid-Positive Allosteric Modulator, and Evaluation of the Bio-Distribution in Non-Human Primate Brains Using Positron Emission Tomography
Source: Molecules. 2024 Jan 15;29(2):427. doi: 10.3390/molecules29020427 (PMC10818776; doi:10.3390/molecules29020427)

Supplementary file

HPLC co-injection method is a technique commonly used to confirm the identity of a radioligand through high-performance liquid chromatography (HPLC). In the co-injection method, a known reference standard and the radioligand are simultaneously injected into an HPLC system. The HPLC system consists of a mobile phase (solvents), a stationary phase (column), and a detection system. The detection system, typically a radioactivity detector or a UV detector, measures the amount of radioactivity or the absorbance of light, respectively, as the separated components elute from the column. By comparing the retention time of the reference standard and the radioligand, their identity can be confirmed. If the retention times of the reference standard and the radioligand are identical, it indicates that they have very similar or identical chemical properties. This confirms that the radioligand being analyzed is indeed the desired compound. This method is essential in quality control and research settings, ensuring the reliability and validity of radioligand studies.

Figure S1: Analytical HPLC chromatogram for the QC of [^{11}C]BIIB104. The upper represents the UV and the lower represents the radio chromatogram.

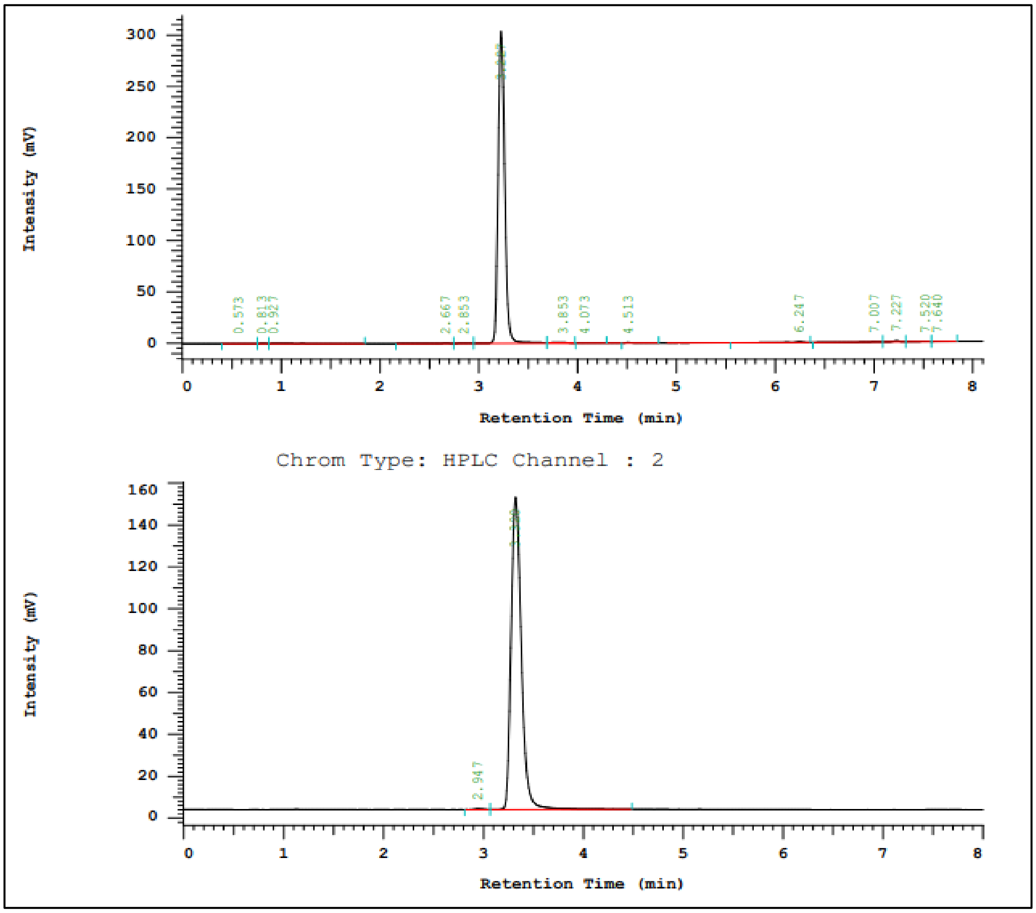

Figure S2: Semi-preparative HPLC chromatogram for the purification of [^{11}C]BIIB104. Upper represents the UV, the middle represents the radio chromatogram and the bottom represents the fraction corresponds to [^{11}C]BIIB104 collected from HPLC

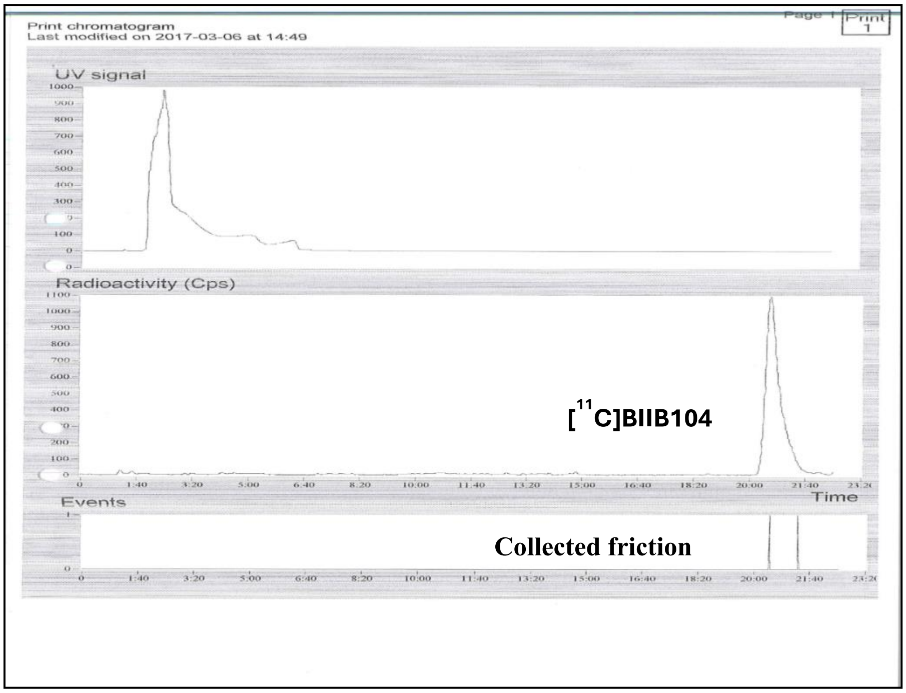

Supplement: Supplementary file 1 [file molecules-29-00427-s001.zip › molecules-2772708-supplementary.pdf]
